# Localized Eosinophilic Ileitis Presenting As Refractory Pediatric Intestinal Pseudo-Obstruction With Recurrent Ileostomy Prolapse: A Delayed Histopathologic Diagnosis

**DOI:** 10.7759/cureus.113412

**Published:** 2026-07-26

**Authors:** Ian L Cummings-Ruiz, Victor Ortiz Justiniano

**Affiliations:** 1 General Surgery, Centro Médico Episcopal San Lucas, Ponce, USA; 2 Surgery, Auxilio Pediátrico, Hospital Auxilio Mutuo, San Juan, PRI

**Keywords:** eosinophilic gastrointestinal disease, eosinophilic ileitis, gastrointestinal dysmotility, ileostomy prolapse, pediatric intestinal pseudo-obstruction, peripheral eosinophilia

## Abstract

Pediatric intestinal pseudo-obstruction (PIPO) is a rare, severe gastrointestinal motility disorder characterized by recurrent symptoms of bowel obstruction in the absence of a mechanical cause. Although most pediatric cases are attributed to primary neuromuscular abnormalities, secondary inflammatory disorders should be considered when symptoms are progressive, refractory to conventional therapy, or associated with peripheral eosinophilia and atopic disease. Localized eosinophilic ileitis is an uncommon manifestation of eosinophilic gastrointestinal disease (EGID) and rarely presents as secondary PIPO, making diagnosis particularly challenging.

We describe a four-year-old Hispanic boy with a history of egg allergy, atopic dermatitis, lactose intolerance, chronic constipation, progressive abdominal distension, anemia, and peripheral eosinophilia who developed recurrent intestinal pseudo-obstruction beginning at two years of age. Hirschsprung disease was excluded by both rectal suction biopsy and subsequent full-thickness rectal biopsy, each demonstrating normal ganglion cells. Persistent symptoms despite maximal medical therapy prompted creation of an end ileostomy for bowel decompression, which required conversion to a loop ileostomy after early prolapse. During the following two years, the patient experienced recurrent ileostomy prolapse requiring multiple operative revisions. Previous surgical specimens demonstrated ischemic and inflammatory changes without eosinophilic infiltration.

Because of persistent obstructive symptoms and recurrent prolapse, definitive ileostomy reversal was performed. Intraoperatively, the prolapsed ileal segment appeared erythematous, mildly thickened, and friable, while the remaining bowel appeared grossly normal. Histopathologic examination of the resected specimen demonstrated focal patchy intramucosal eosinophilia exceeding 70 eosinophils per high-power field, establishing the diagnosis of localized eosinophilic ileitis after exclusion of secondary infectious causes. No eosinophilic infiltration had been identified in previous rectal or ileostomy specimens.

The patient experienced rapid postoperative recovery and remains asymptomatic 12 months after surgery without recurrent pseudo-obstruction or prolapse. This case highlights the diagnostic challenges posed by localized eosinophilic ileitis, demonstrates the limitations of negative biopsies obtained from uninvolved bowel, and emphasizes the importance of considering EGID in children with refractory intestinal dysmotility, peripheral eosinophilia, and atopic disease.

## Introduction

Pediatric intestinal pseudo-obstruction (PIPO) is one of the most complex gastrointestinal motility disorders encountered in pediatric practice. It is characterized by recurrent episodes of bowel obstruction in the absence of an anatomic occlusion and results from impaired intestinal propulsion caused by abnormalities affecting the enteric nervous system, intestinal smooth muscle, interstitial cells of Cajal, or secondary inflammatory processes. Although uncommon, PIPO carries substantial morbidity because affected children frequently experience repeated hospitalizations, prolonged nutritional compromise, multiple operative interventions, and delayed diagnosis. Current consensus recommendations emphasize the importance of systematically excluding structural, neuromuscular, metabolic, infectious, autoimmune, and genetic etiologies before establishing the diagnosis of primary PIPO [1,2].

While primary neuromuscular disorders account for most pediatric cases, secondary causes are increasingly recognized and should not be overlooked, particularly when the clinical course is atypical. Inflammatory disorders involving the bowel wall may disrupt intestinal motility through direct injury to the enteric nervous system or intestinal musculature, producing symptoms that closely resemble primary intestinal pseudo-obstruction. Among these disorders, eosinophilic gastrointestinal disease (EGID) represents a rare but important consideration because its presentation is highly variable and often nonspecific [3].

EGIDs comprise a spectrum of chronic inflammatory disorders characterized by pathologic eosinophilic infiltration of one or more segments of the gastrointestinal tract after exclusion of secondary causes of tissue eosinophilia, including parasitic infection, inflammatory bowel disease, vasculitis, connective tissue disorders, drug reactions, and malignancy. Clinical manifestations depend on the location and depth of bowel involvement and may include abdominal pain, nausea, vomiting, diarrhea, constipation, anemia, protein-losing enteropathy, intestinal obstruction, or failure to thrive [4,5]. Although eosinophilic esophagitis is well recognized, eosinophilic involvement of the small intestine is considerably less common, and isolated eosinophilic ileitis remains an exceptionally rare diagnosis in children [6].

The diagnosis of eosinophilic ileitis presents several challenges. Unlike eosinophilic esophagitis, there are no universally accepted histopathologic thresholds for disease throughout all segments of the gastrointestinal tract, and eosinophilic infiltration is frequently patchy rather than diffuse. Consequently, endoscopic or surgical biopsies obtained from uninvolved bowel may be nondiagnostic despite active disease elsewhere. In addition, many patients present with overlapping features of more common pediatric gastrointestinal disorders, including Hirschsprung disease, inflammatory bowel disease, food protein-induced enterocolitis, and functional gastrointestinal disorders, often resulting in prolonged diagnostic evaluation before the underlying inflammatory process is recognized [4,7].

Accumulating evidence suggests that eosinophilic inflammation may contribute to gastrointestinal dysmotility through multiple mechanisms. Activated eosinophils release cytotoxic granule proteins, cytokines, and inflammatory mediators capable of disrupting smooth muscle function, altering enteric neuronal signaling, and impairing the activity of interstitial cells of Cajal, thereby producing clinical manifestations that mimic chronic intestinal pseudo-obstruction [8]. Although this association has been described in isolated case reports and small series, localized eosinophilic ileitis presenting as refractory PIPO remains exceedingly uncommon.

We report the case of a four-year-old boy with progressive intestinal pseudo-obstruction, recurrent ileostomy prolapse, peripheral eosinophilia, and atopic disease whose diagnosis of localized eosinophilic ileitis was established only after definitive surgical resection. This case underscores the importance of maintaining a broad differential diagnosis in children with refractory gastrointestinal dysmotility and illustrates how localized disease and sampling error may substantially delay recognition of EGID despite multiple previous biopsies and surgical interventions.

## Case presentation

A four-year-old Hispanic boy was referred to our institution for evaluation of recurrent intestinal pseudo-obstruction after a prolonged history of progressive constipation, abdominal distension, recurrent vomiting, and multiple ileostomy complications. His medical history was significant for mild egg allergy with rash, atopic dermatitis during infancy, lactose intolerance, chronic microcytic anemia requiring iron supplementation, and persistent peripheral eosinophilia. Pregnancy was complicated by maternal preeclampsia, prompting cesarean delivery at 39 weeks' gestation. Birth weight was 3.66 kg, and meconium was passed within the first 24 hours of life. Early infancy and developmental milestones were otherwise unremarkable.

The family history was notable for chronic constipation in the patient's mother and neonatal Hirschsprung disease requiring colostomy in a maternal cousin. Additional maternal relatives had histories of diverticular disease, colitis, intestinal obstruction, and other gastrointestinal disorders. There was no known family history of inherited neuromuscular disorders or chronic intestinal pseudo-obstruction.

At approximately two years and four months of age, the patient developed intermittent constipation associated with progressive abdominal distension. Symptoms persisted despite treatment with polyethylene glycol and simethicone. His diet gradually became restricted because oral intake worsened abdominal discomfort and distension. Over the following months, he developed recurrent episodes of non-bilious emesis, progressive pallor, and iron-deficiency anemia requiring hematology evaluation and oral iron supplementation.

**Figure 1:**
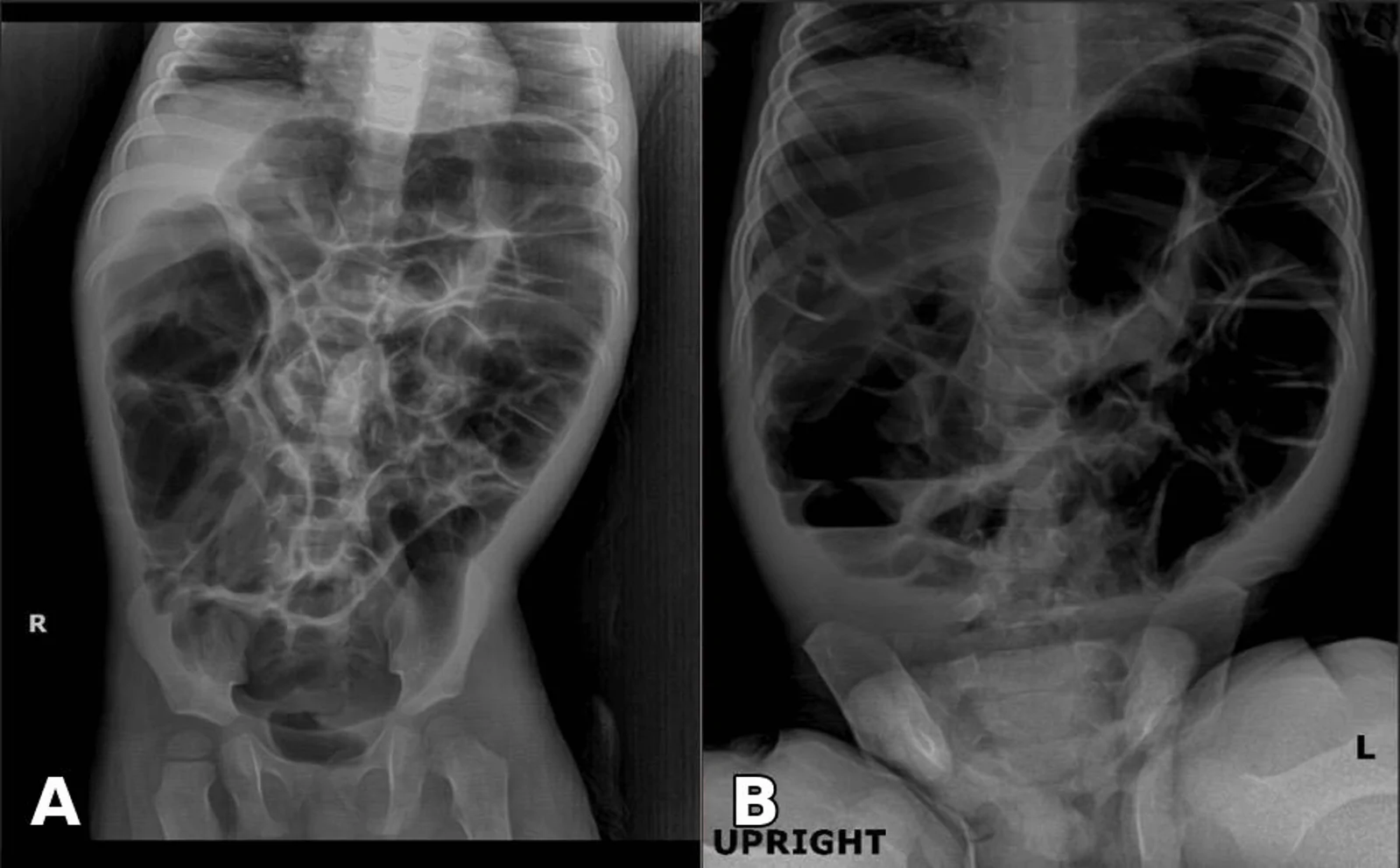
Abdominal radiographs demonstrating diffuse bowel dilation consistent with pseudo-obstruction on index admission. (A) Supine abdominal radiograph demonstrating diffuse gaseous dilation of multiple bowel loops without evidence of pneumoperitoneum or a discrete mechanical obstruction. (B) Upright abdominal radiograph demonstrating multiple dilated bowel loops with air-fluid levels in the absence of a mechanical obstruction.

During his initial hospitalization at an outside institution, abdominal radiographs demonstrated diffuse bowel dilation without evidence of a mechanical obstruction (Figure 1). Because Hirschsprung disease remained a major diagnostic consideration, a rectal suction biopsy was performed and demonstrated normal ganglion cells. Persistent obstructive symptoms despite medical management prompted a subsequent full-thickness rectal biopsy obtained through rectal myomectomy, which again demonstrated ganglion cells without evidence of aganglionosis or eosinophilic infiltration. The postoperative course was complicated by a recurrent perirectal abscess requiring operative drainage and placement of a central venous catheter for nutritional support.

Despite exclusion of Hirschsprung disease, the patient's symptoms progressively worsened. During the same hospitalization, an end ileostomy was created for bowel decompression because of medically refractory intestinal pseudo-obstruction. On postoperative day 1, early stoma prolapse required conversion to a loop ileostomy.

Following diversion, erythromycin was initiated as a prokinetic agent, resulting in temporary symptomatic improvement. Additional medications administered throughout his clinical course included cyproheptadine, polyethylene glycol, simethicone, oral iron supplementation, Intestinex, and intermittent doxycycline.

Over the following two years, the patient experienced multiple episodes of recurrent ileostomy prolapse requiring repeated emergency department visits and several operative revisions. Conservative reduction techniques, including topical granulated sugar, were attempted but frequently failed. Histopathologic examination of previous ileostomy specimens demonstrated ischemic changes, edema, congestion, mucosal ulceration, and acute suppurative inflammation without eosinophilic infiltration. Ganglion cells were identified in all specimens.

Approximately two years after ileostomy creation, the patient was readmitted with severe abdominal pain, progressive abdominal distension, recurrent non-bilious emesis, pallor, dizziness, near-syncope, and recurrent ileostomy prolapse.

Laboratory evaluation demonstrated leukocytosis, microcytic anemia, thrombocytosis, absolute eosinophilia, mild metabolic acidosis, elevated inflammatory markers, and mildly elevated serum IgE (Table 1). Initial IgE testing performed at the referring institution had reportedly been normal. Stool studies and parasite serologies were negative, and thyroid function tests were within normal limits. According to the patient's mother, comprehensive genetic testing performed at another institution had reportedly yielded negative results; however, the original report was unavailable for review.

**Table 1:** Pertinent laboratory findings during definitive hospitalization.

Parameter	Patient Value	Reference Range
White blood cell count	12.18 × 10⁹/L	3.98-10.04 × 10⁹/L
Mean corpuscular volume (MCV)	67.6 fL	79.0-94.8 fL
Mean corpuscular hemoglobin (MCH)	22.1 pg	25.6-32.2 pg
Red cell distribution width (RDW)	25.0%	11.6-14.4%
Platelet count	743 × 10⁹/L	163-369 × 10⁹/L
Absolute eosinophil count	2.38 × 10⁹/L	0.04-0.54 × 10⁹/L
Eosinophils	19.5%	0.7-7.0%
Carbon dioxide (CO₂)	17 mmol/L	22-29 mmol/L
Anion gap	24 mmol/L	12-20 mmol/L
C-reactive protein	6 mg/L	0-3 mg/L
Erythrocyte sedimentation rate	21 mm/hr	0-13 mm/hr
Serum IgE	102 IU/mL	0-60 IU/mL

A water-soluble contrast enema demonstrated a partially collapsed colon without evidence of a transition zone or intrinsic obstruction. Repeat multidirectional contrast studies performed through the oral route, nasogastric tube, ileostomy, and rectum demonstrated functional obstruction caused by angulation and kinking of the ileostomy rather than an intrinsic bowel obstruction. Upper gastrointestinal endoscopy and colonoscopy with terminal ileal biopsies were not performed during the diagnostic evaluation.

Because of recurrent prolapse, persistent obstructive symptoms, and failure of multiple previous operative revisions, definitive ileostomy takedown was recommended during the same hospitalization. Intraoperatively, the prolapsed ileal segment appeared erythematous, mildly thickened, and friable, whereas the remaining small bowel and colon appeared grossly viable without diffuse inflammatory disease or intrinsic obstructing lesions. Approximately 7 cm of abnormal ileum adjacent to the stoma was resected, followed by restoration of intestinal continuity with ileocolic anastomosis.

Histopathologic examination demonstrated serosal fibrotic adhesions associated with acute, subacute, and chronic inflammation, foreign-body granulomas, severe ischemic changes involving the prolapsed stoma, mucosal ulceration, granulation tissue, fistulous tract formation, and focal patchy intramucosal eosinophilia exceeding 70 eosinophils per high-power field. Histopathological slides are, however, unavailable. No microorganisms were identified. In the clinical context of peripheral eosinophilia, atopic disease, and negative infectious studies, the findings were consistent with localized eosinophilic ileitis.

The postoperative course was uncomplicated. The patient passed flatus on postoperative day 1, advanced from clear liquids to a regular diet over the following 48 hours, and resumed spontaneous bowel movements shortly thereafter. Because the diagnosis was established only after surgical resection and symptoms resolved completely, corticosteroids, dietary elimination therapy, and biologic treatment were deferred. At 12-month follow-up, he remained asymptomatic, tolerated a regular diet, and had experienced no recurrence of abdominal distension, vomiting, intestinal pseudo-obstruction, or ileostomy-related complications.

## Discussion

PIPO is among the most challenging disorders encountered by pediatric gastroenterologists and surgeons because it represents a clinical syndrome rather than a single disease entity. Children typically present with recurrent symptoms of intestinal obstruction in the absence of a mechanical lesion, requiring an extensive evaluation to distinguish primary enteric neuromuscular disorders from secondary causes of dysmotility. Although congenital neuropathies and myopathies account for most pediatric cases, inflammatory, autoimmune, infectious, metabolic, and genetic disorders should be considered when the clinical course is atypical or refractory to conventional management [1,2].

Our patient presented with several clinical features that complicated the diagnostic process. Severe constipation, progressive abdominal distension, recurrent vomiting, and diffuse bowel dilation initially raised concern for Hirschsprung disease, one of the most common causes of lower intestinal obstruction in young children. Although passage of meconium within the first 24 hours of life made classic Hirschsprung disease less likely, normal neonatal stool passage does not completely exclude short-segment disease or atypical presentations. Accordingly, rectal suction biopsy followed by full-thickness rectal biopsy represented an appropriate diagnostic strategy. Both specimens demonstrated ganglion cells without evidence of aganglionosis, effectively excluding Hirschsprung disease while also demonstrating no evidence of eosinophilic infiltration [3].

One of the most instructive aspects of this case is the prolonged delay before the diagnosis of eosinophilic ileitis was established. The patient underwent multiple surgical procedures over nearly two years, including ileostomy creation, conversion to a loop ileostomy, repeated prolapse revisions, and segmental bowel resections before eosinophilic infiltration was finally identified. Importantly, previous pathologic specimens consistently demonstrated ischemic and inflammatory changes associated with recurrent prolapse but did not report eosinophilic infiltration. Rather than representing a missed diagnosis by the pathologist, these findings likely reflect the localized and patchy distribution characteristic of EGID. Multiple studies have demonstrated that eosinophilic inflammation may involve isolated bowel segments while sparing adjacent tissue, making diagnostic yield highly dependent on biopsy location [4,5]. Consequently, negative pathology from one gastrointestinal segment should not exclude EGID when the overall clinical picture remains suspicious.

In retrospect, several clinical findings supported an inflammatory rather than purely neuromuscular process. The patient had a well-documented history of egg allergy, infantile atopic dermatitis, lactose intolerance, persistent peripheral eosinophilia, chronic microcytic anemia, and mildly elevated serum IgE. Although none of these findings independently establishes the diagnosis of EGID, their coexistence substantially increases clinical suspicion, particularly when associated with unexplained gastrointestinal dysmotility [4,6]. Secondary causes of eosinophilia were appropriately investigated. Stool studies and parasite serologies were negative, thyroid function testing was normal, and no microorganisms were identified on histopathologic examination. Initial IgE testing reportedly performed at the referring institution had been normal, whereas repeat testing at our institution demonstrated only mild elevation. This observation highlights an important clinical point: serum IgE lacks sufficient sensitivity or specificity to either confirm or exclude EGID and should not be used as an isolated diagnostic marker [4].

The absence of upper gastrointestinal endoscopy and colonoscopy with terminal ileal biopsies deserves particular attention. Current consensus recommendations emphasize that endoscopic biopsy remains the cornerstone of diagnosis for EGID whenever clinically feasible [4]. However, because eosinophilic inflammation frequently demonstrates a discontinuous distribution, even extensive biopsy protocols may fail to identify localized disease. In our patient, earlier ileal biopsies obtained during colonoscopy might have established the diagnosis before repeated surgical interventions became necessary. Conversely, the disease may have remained undetected if uninvolved bowel had been sampled. This limitation illustrates the inherent diagnostic difficulty of localized eosinophilic ileitis and should be considered whenever clinical suspicion remains high despite nondiagnostic pathology.

The radiologic evaluation also warrants discussion because several reviewers questioned the rationale for repeated contrast studies. A water-soluble contrast enema was performed to evaluate the distal bowel before consideration of ileostomy reversal and to exclude a distal mechanical obstruction or transition zone. Subsequent multidirectional contrast studies administered through the oral route, nasogastric tube, ileostomy, and rectum demonstrated functional obstruction caused by angulation and kinking of the ileostomy rather than intrinsic bowel obstruction. These findings explained the patient's recurrent obstructive episodes but did not account for the underlying inflammatory process, ultimately emphasizing that radiologic studies alone cannot distinguish primary dysmotility from localized inflammatory disease.

The indication for definitive surgery also deserves clarification. Ileostomy reversal was not undertaken because eosinophilic ileitis was suspected. Rather, surgery was indicated because of recurrent prolapse, persistent obstructive symptoms, and failure of multiple previous operative revisions. At exploration, the prolapsed ileal segment appeared erythematous, mildly thickened, and friable, whereas the remaining small bowel and colon appeared grossly normal. These findings justified limited resection of the visibly abnormal bowel immediately adjacent to the stoma. Frozen sections were not performed, and the resection margins were not evaluated histologically for eosinophilic infiltration because the diagnosis was not suspected intraoperatively. Histopathologic examination of the resected specimen subsequently demonstrated focal patchy intramucosal eosinophilia exceeding 70 eosinophils per high-power field, establishing the diagnosis of localized eosinophilic ileitis.

It is important to emphasize that surgery is not considered first-line therapy for EGID. Current management strategies favor dietary modification and corticosteroid therapy as initial treatment, while biologic agents and other immunomodulatory therapies are increasingly used for refractory disease [4,7]. Surgical intervention is generally reserved for complications including perforation, ischemia, strictures, obstruction, intussusception, or failure of conservative therapy. In the present case, surgery was performed to address recurrent ileostomy prolapse and persistent functional obstruction rather than to treat eosinophilic ileitis. The diagnosis was established only after histopathologic examination of the resected specimen. Therefore, the favorable postoperative outcome should not be interpreted as evidence that surgical resection alone cures EGID. Instead, resection removed the localized diseased bowel responsible for the patient's symptoms while simultaneously establishing the diagnosis. Ongoing gastroenterology follow-up remains essential because recurrence within other gastrointestinal segments remains possible.

Another important differential diagnosis in children with severe early-onset intestinal dysmotility is primary genetic PIPO. Pathogenic variants involving genes such as ACTG2, FLNA, and MYH11 have been associated with congenital visceral myopathy and chronic intestinal pseudo-obstruction [8]. According to the patient's mother, comprehensive genetic testing had previously been performed at the referring institution and reportedly yielded negative results; however, those records were unavailable for independent review. Although this limits complete interpretation of the diagnostic evaluation, the patient's localized histopathologic findings, peripheral eosinophilia, atopic history, and sustained clinical improvement after resection strongly support a secondary inflammatory process rather than a primary inherited motility disorder.

The association between eosinophilic inflammation and gastrointestinal dysmotility has become increasingly recognized over the past decade. Experimental studies suggest that activated eosinophils release major basic protein, eosinophil-derived neurotoxin, cytokines, and profibrotic mediators capable of disrupting smooth muscle contractility, altering enteric neuronal signaling, and impairing the function of interstitial cells of Cajal. These mechanisms provide biologic plausibility for the severe dysmotility observed in our patient despite the absence of an intrinsic mechanical obstruction. Although muscularis propria involvement could not be fully assessed histologically in this case, the clinical presentation and sustained improvement following resection support eosinophilic ileitis as the most plausible secondary contributor to the patient's prolonged intestinal dysmotility.

Several limitations should be acknowledged. Serial anthropometric measurements from the referring institution were unavailable, precluding formal assessment of growth velocity and nutritional status throughout the disease course. Nutritional laboratory markers, including albumin, prealbumin, ferritin, and celiac serologies, were not consistently available. Upper gastrointestinal endoscopy and colonoscopy with terminal ileal biopsies were not performed before surgery, preventing assessment for more extensive eosinophilic gastrointestinal involvement, and histopathological slides were unavailable upon request. Finally, although the patient remains asymptomatic 12 months after surgery, longer follow-up will be necessary because EGID is a chronic inflammatory disorder with potential for recurrence.

Despite these limitations, this case contributes to the limited literature describing localized eosinophilic ileitis presenting as secondary PIPO. More importantly, it illustrates how repeated negative biopsies do not exclude EGID when tissue is obtained from uninvolved bowel. Clinicians should maintain a high index of suspicion for EGID in children with chronic intestinal dysmotility accompanied by atopy, peripheral eosinophilia, unexplained anemia, or recurrent obstructive symptoms despite appropriate evaluation for Hirschsprung disease and other structural abnormalities. Earlier recognition and targeted biopsy of affected bowel segments may shorten the diagnostic course and facilitate timely institution of appropriate medical therapy before repeated operative interventions become necessary.

## Conclusions

Localized eosinophilic ileitis is an uncommon manifestation of EGID and should be considered in the differential diagnosis of children with refractory PIPO, particularly when accompanied by atopic disease, peripheral eosinophilia, or unexplained anemia. This case illustrates how localized disease and sampling error can delay diagnosis despite multiple previous biopsies and operative interventions. Repeated negative rectal and ileostomy specimens did not exclude EGID because earlier biopsies sampled uninvolved bowel. The diagnosis was ultimately established only after histopathologic examination of the grossly abnormal ileal segment resected during surgery for recurrent ileostomy prolapse and persistent functional obstruction. This emphasizes the importance of routine histopathologic evaluation of all resected bowel specimens and careful clinicopathologic correlation in children with unexplained gastrointestinal dysmotility. It is also important to note that unexplained peripheral eosinophilia may be a potential clinical trigger for endoscopic evaluation in these patients.

Current management remains centered on dietary modification and anti-inflammatory therapy, with operative intervention reserved for complications or medically refractory disease. In our patient, surgery addressed recurrent prolapse and obstruction while simultaneously providing the tissue necessary to establish the diagnosis. The patient remains asymptomatic 12 months after surgery; however, continued gastroenterology follow-up is warranted because EGID is chronic and recurrence remains possible. Earlier recognition of EGID through targeted biopsy of affected bowel segments may reduce diagnostic delays and potentially prevent repeated surgical interventions in selected patients.

## References

[1] Thapar N, Saliakellis E, Benninga MA (2018). Paediatric intestinal pseudo-obstruction: evidence and consensus-based recommendations from an ESPGHAN-led expert group. J Pediatr Gastroenterol Nutr.

[2] Turcotte MC, Faure C (2022). Pediatric intestinal pseudo-obstruction: progress and challenges. Front Pediatr.

[3] Papadopoulou A, Amil-Dias J, Auth MK (2024). Joint ESPGHAN/NASPGHAN guidelines on childhood eosinophilic gastrointestinal disorders beyond eosinophilic esophagitis. J Pediatr Gastroenterol Nutr.

[4] Papaiakovou G, Papageorgiou A, Bakakos A, Sinaniotis AC, Rovina N (2024). Eosinophilic gastrointestinal diseases: current perspectives on pathogenesis and management. Explor Allergy Asthma.

[5] Collins MH (2014). Histopathologic features of eosinophilic esophagitis and eosinophilic gastrointestinal diseases. Gastroenterol Clin North Am.

[6] Ghosh S, Hiwale KM (2024). Challenges in diagnosis and management of unusual cases of eosinophilic enteritis in rural health settings: a comprehensive review. Cureus.

[7] Rothenberg ME (2004). Eosinophilic gastrointestinal disorders (EGID). J Allergy Clin Immunol.

[8] Ooms AH, Verheij J, Hulst JM, Vlot J, van der Starre C, de Ridder L, de Krijger RR (2012). Eosinophilic myenteric ganglionitis as a cause of chronic intestinal pseudo-obstruction. Virchows Arch.

